# Supplementary figures and images for: SPARC Promotes Cell Invasion In Vivo by Decreasing Type IV Collagen Levels in the Basement Membrane
Source: PLoS Genet. 2016 Feb 29;12(2):e1005905. doi: 10.1371/journal.pgen.1005905 (PMC4771172; doi:10.1371/journal.pgen.1005905)

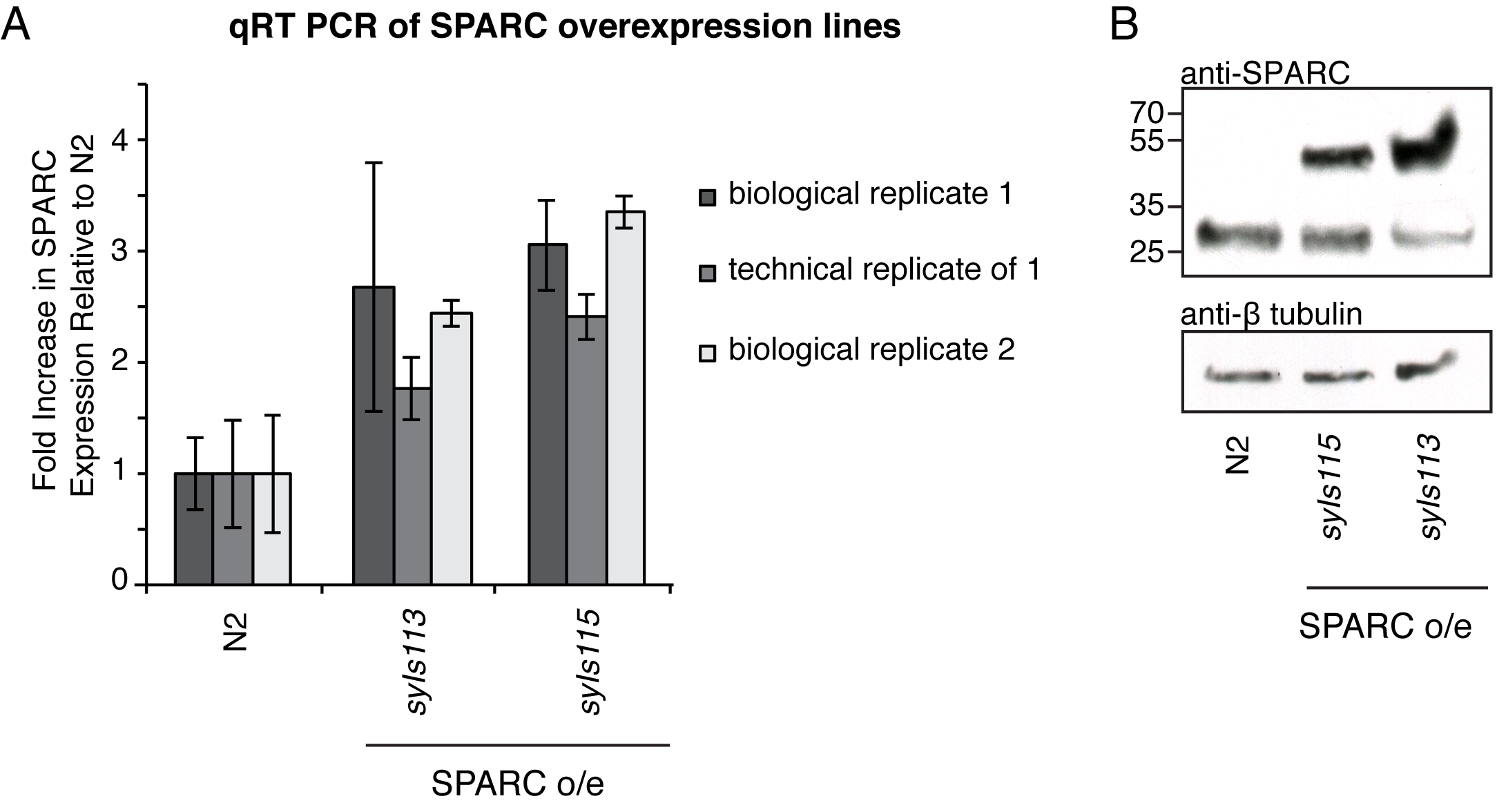

Supplement: S1 Fig — (A) Quantitative Real Time PCR of SPARC levels in wild type animals and animals expressing integrated transgenes overexpressing SPARC::GFP (syIs113 and syIs115). SPARC mRNA levels were standardized using act-4 (actin) mRNA as a control and normalized to wild type (N2) levels. Error bars denote standard deviation in each of three replicates of the RT-PCR reaction. (B) A representative western blot shows endogenous SPARC (27 kD) and overexpressed SPARC::GFP (54 kD) in sysIs115 and syIs113 transgenic lines. An average of three western blots showed that syIs115 animals express 2.5±0.4 fold more SPARC protein relative to endogenous SPARC and syIs113 animals have a 5.4±1.6 fold increase in SPARC protein expression. (TIF) [file pgen.1005905.s001.tif]

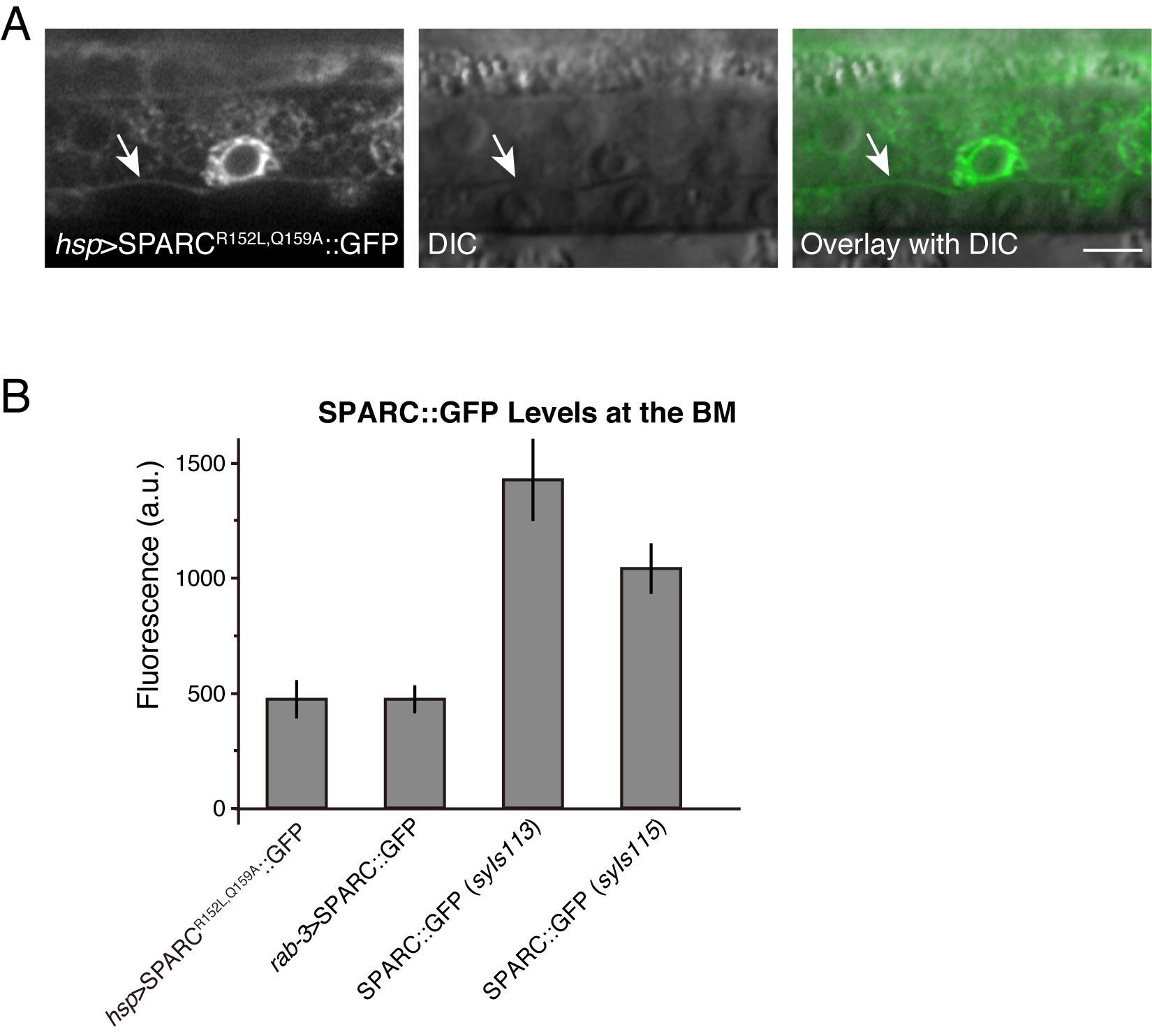

Supplement: S2 Fig — (A) hsp>SPARCR152L,Q159A::GFP (green, left; overlaid with DIC, right) is found in the BM (arrows). (B) Quantification of SPARC::GFP fluorescence at the BM in SPARC overexpression lines. As SPARC overexpression does not appear to affect the expression of endogenous unlabeled SPARC (see S1 Fig), these graphs depict only the population of SPARC present in excess of endogenous levels. Error bars denote SEM. Scale bars denote 5 μm. (TIF) [file pgen.1005905.s002.tif]

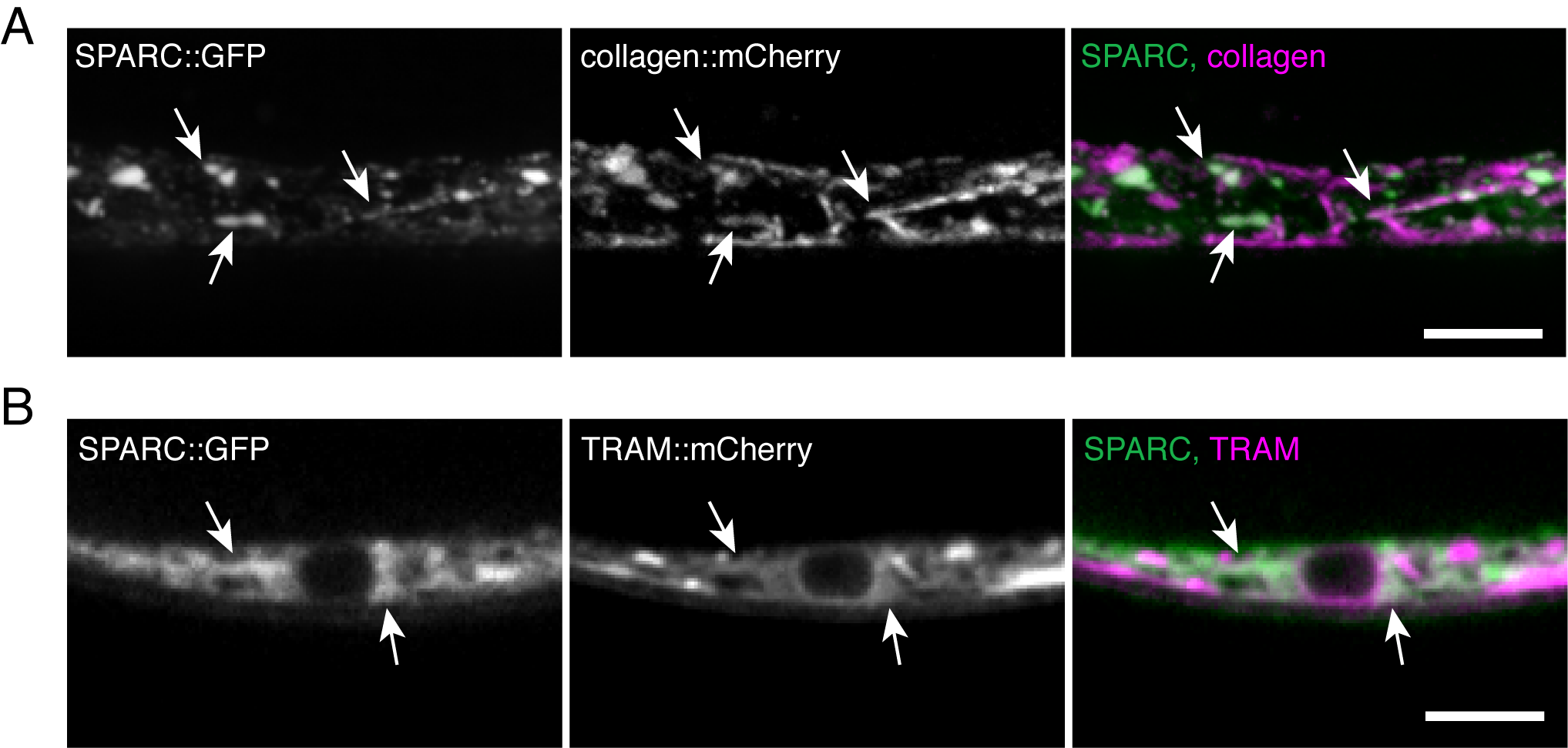

Supplement: S3 Fig — (A) SPARC::GFP (syIs115 line; left) and collagen::mCherry (center) colocalize in vesicles within the body wall muscle (overlay, right; average Pearson’s correlation coefficient = 0.70 ± 0.01; n = 8 animals). (B) Overexpressed SPARC::GFP (syIs115; left) is predominantly localized in the rough ER (as marked with TRAM::mCherry, middle; overlay, right; n = 5 animals). Arrows highlight regions of colocalization. (TIF) [file pgen.1005905.s003.tif]

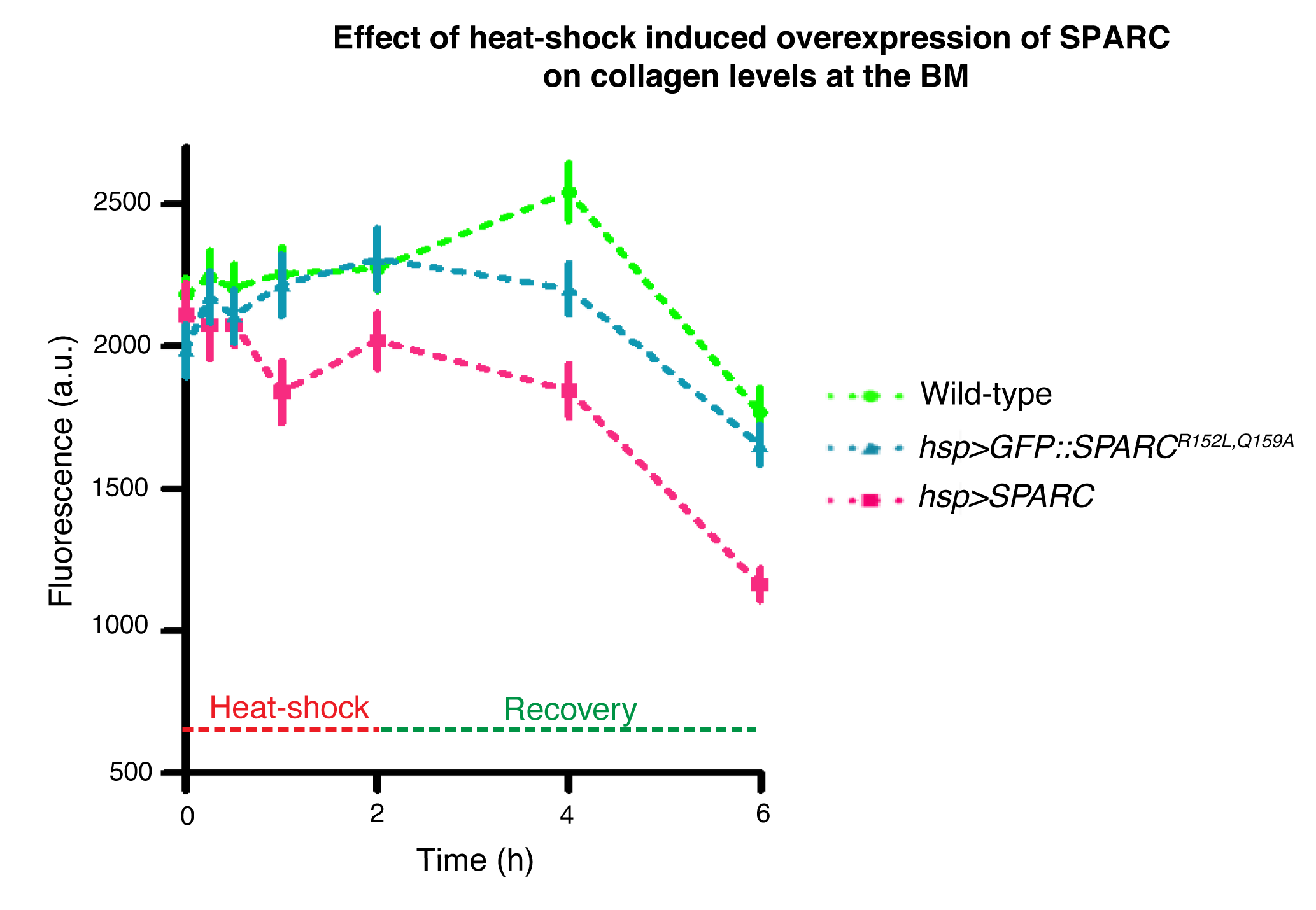

Supplement: S4 Fig — Worms expressing hsp>SPARC, hsp>SPARCR152L,Q159A::GFP, or no excess SPARC were subjected to a two hour heat shock at 32°C to drive SPARC overexpression, followed by four hours of recovery at 23°C. Collagen::mCherry fluorescence at the BM was measured at the indicated time points after heat shock was initiated (n≥20 animals for each treatment at each timepoint). Error bars denote SEM. (TIF) [file pgen.1005905.s004.tif]

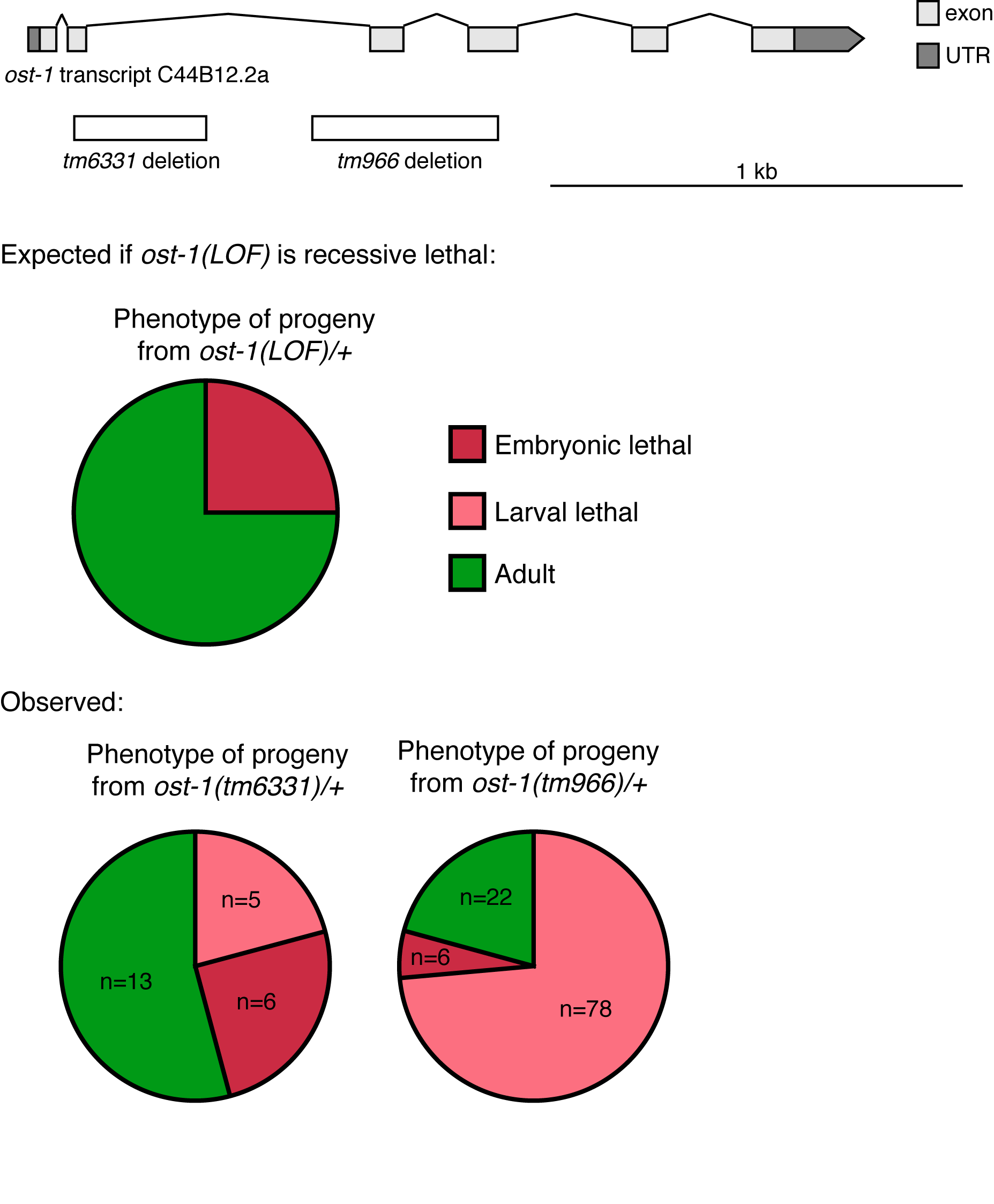

Supplement: S5 Fig — Two non-overlapping deletions in the SPARC open reading frame (ost-1) were obtained from the C. elegans Gene Knockout Consortium. The location of the deletions tm6331 and tm966 is shown on top. Below, the progeny from a timed egg lay of ost-1(tm6331)/+ (left) or ost-1(tm966)/+ (right). The progeny of the egg lay were tracked for one week and their phenotypes were recorded. More than 25% of the progeny of ost-1(tm6331) and ost-1(tm966) did not survive to adulthood (green), suggesting that homozygotes of both mutant alleles are embryonic (dark red) or larval (light red) lethal. (TIF) [file pgen.1005905.s005.tif]
